# Parallel β-Sheet Structure and Structural Heterogeneity Detected within Q11 Self-Assembling Peptide Nanofibers

**DOI:** 10.1021/acs.jpcb.4c00825

**Published:** 2024-05-24

**Authors:** Alicia S. Robang, Kong M. Wong, Johannes Leisen, Renjie Liu, Walker L. Radford, Tarunya Rao Sudarshan, Gregory A. Hudalla, Anant K. Paravastu

**Affiliations:** † School of Chemical and Biomolecular Engineering, Georgia Institute of Technology, Atlanta, Georgia 30332, United States; ‡ School of Chemistry & Biochemistry, Georgia Institute of Technology, Atlanta, Georgia 30332, United States; § J. Crayton Pruitt Family Department of Biomedical Engineering, University of Florida, Gainesville, Florida 32611, United States; ∥ Parker H. Petit Institute for Bioengineering and Biosciences, Georgia Institute of Technology, Atlanta, Georgia 30332, United States

## Abstract

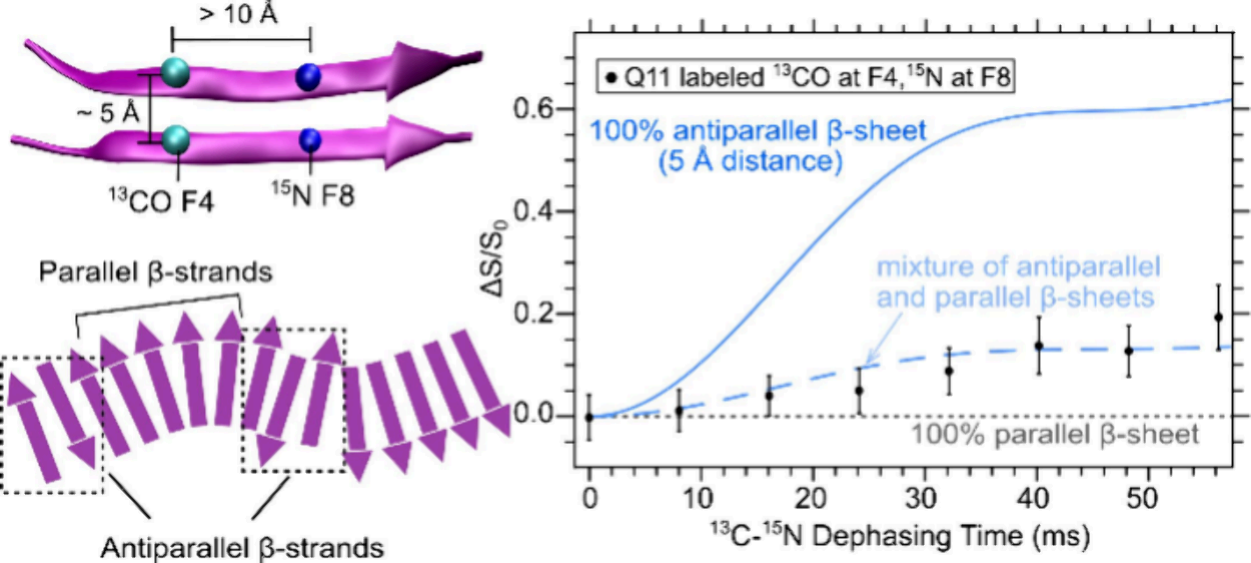

Q11 peptide nanofibers are used as a biomaterial for applications such as antigen presentation and tissue engineering, yet detailed knowledge of molecular-level structure has not been reported. The Q11 peptide sequence was designed using heuristics-based patterning of hydrophobic and polar amino acids with oppositely charged amino acids placed at opposite ends of the sequence to promote antiparallel β-sheet formation. In this work, we employed solid-state nuclear magnetic resonance spectroscopy (NMR) to evaluate whether the molecular organization within Q11 self-assembled peptide nanofibers is consistent with the expectations of the peptide designers. We discovered that Q11 forms a distribution of molecular structures. NMR data from two-dimensional (2D) ^13^C–^13^C dipolar-assisted rotational resonance indicate that the K3 and E9 residues between Q11 β-strands are spatially proximate (within ∼0.6 nm). Frequency-selective rotational echo double resonance (fsREDOR) on K3 Nζ and E9 Cδ-labeled sites showed that approximately 9% of the sites are close enough for salt bridge formation to occur. Surprisingly, dipolar recoupling measurements revealed that Q11 peptides do not assemble into antiparallel β-sheets as expected, and structural analysis using Fourier-transform infrared spectroscopy and 2D NMR alone can be misleading. ^13^C PITHIRDS-CT dipolar recoupling measurements showed that the most abundant structure consists of parallel β-sheets, in contrast to the expected antiparallel β-sheet structure. Structural heterogeneity was detected from ^15^N{^13^C} REDOR measurements, with approximately 22% of β-strands having antiparallel nearest neighbors. We cannot propose a complete structural model of Q11 nanofibers because of the complexity involved when examining structurally heterogeneous samples using NMR. Altogether, our results show that while heuristics-based patterning is effective in promoting β-sheet formation, designing a peptide sequence to form a targeted β-strand arrangement remains challenging.

## Introduction

Q11 (Ac-QQKFQFQFEQQ-Am) peptide-based nanofibers have been investigated as a biomaterial for therapeutic applications. However, the nanofiber molecular structure has not yet been reported. Q11 was developed by Collier et al. as a transglutaminase substrate for lysine-containing biomolecules.^1^ Subsequent studies have shown that Q11 peptides can be modified or conjugated with other biomolecules for applications such as vaccine delivery and extracellular scaffold mimicry in regenerative medicine.^2−6^ The amino acid sequence of Q11 includes alternating hydrophobic and polar amino acids to promote β-strand secondary structure. Charged amino acids lysine and glutamic acid are positioned near opposite ends of the peptide sequence to promote an antiparallel β-sheet structure.^7^ Previous biophysical measurements confirmed the formation of β-sheets from self-assembled Q11 peptides. In this study, we are interested in testing whether Q11 effectively assembles into the anticipated β-sheet self-assembled structure.

We report features of the Q11 nanofiber molecular structure probed by solid-state nuclear magnetic resonance (NMR) spectroscopy. Consistent with previous biophysical studies, one-dimensional ^13^C spectra collected from ^1^H–^13^C cross-polarization magic angle spinning (CPMAS) measurements confirm that Q11 peptide solutions form nanofibers composed of β-sheets when prepared in the buffer of 137 NaCl, 2.7 KCl, 10 Na_2_HPO_4_, and 1.8 mM KH_2_PO_4_ (1× phosphate-buffered saline or PBS). 2D ^13^C–^13^C dipolar-assisted rotational resonance (DARR) and frequency-selective rotational echo double resonance REDOR (fsREDOR) measurements show proximity between lysine and glutamic acid residues.^8,9^ Proximity between these residues can be explained either by antiparallel stacking between β-sheet layers or antiparallel β-strand neighbors within β-sheets. Surprisingly, distance-dependent dipolar recoupling NMR measurements (^13^C PITHIRDS-CT and ^15^N{^13^C} rotational echo double resonance (REDOR)) reveal that Q11 nanofibers consist mostly of parallel β-sheets. Structural heterogeneity within Q11 samples is also apparent from the multiple carbonyl peaks observed in the ^13^C spectra of a Q11 sample labeled with ^13^C at a single carbonyl site. NMR also detects a degree of antiparallel organization of adjacent β-strands. Thus, Q11 peptides self-assemble into structurally heterogeneous nanofibers primarily containing parallel β-sheets and not antiparallel β-sheets as anticipated.

## Materials and Methods

### Peptide Synthesis

We synthesized Q11 peptides (Ac-QQKFQFQFEQQ-Am; Ac: acetylated, Am: amidated) using Fmoc solid-phase synthesis on a CEM Liberty Blue Automated Microwave Peptide Synthesizer with a Rink Amide Resin-Protide (CEM Corporation), standard amino acids (Sigma-Aldrich, Aapptec Inc., CEM Corporation). We also synthesized Q11 using isotopically enriched amino acids (Cambridge Isotope Laboratories, Inc.) to produce labeled Q11 samples. To acetylate the N-termini, we used 10% acetic anhydride in dimethylformamide (Sigma-Aldrich). Following synthesis, we cleaved peptides from the resin with a cleavage cocktail of 92.5% trifluoroacetic acid (VWR International), 2.5% triisopropylsilane (Thermo Fisher Scientific), 2.5% 3,6-dioxa-1,8-octanedithiol (VWR International), and 2.5% Milli-Q water. After sitting in the cleavage cocktail for 3 h, we separated the soluble Q11 peptide from the resin using Razor Cleavage Vessels (CEM Corporation) with a 37-μm porosity frit and precipitated the peptide with diethyl ether (VWR International). We then centrifuged the peptide precipitates at 5922 relative centrifugal force (RCF)RCF and 4 °C for 30 min and washed them with fresh diethyl ether one more time to remove residual trifluoroacetic acid (TFA). Finally, we dissolved the peptide in Milli-Q water, froze it in liquid N_2_, and freeze-dried it overnight.

### Peptide Purification

We verified the molecular weights of all peptides using matrix-assisted laser desorption/ionization time-of-flight (MALDI-TOF) mass spectrometry, liquid chromatography–mass spectrometry (LC-MS), or electrospray ionization–mass spectrometry (ESI–MS) conducted by the System Mass Spectrometry Core (SyMS-C) facility at the Georgia Institute of Technology on crude peptides prior to high-performance liquid chromatography (HPLC) purification. We purified Q11 peptide labeled with ^13^CO at F4 and ^15^N at F8 to at least 95% purity using a Dionex Ultimate 3000 system (Thermo Scientific) with a Hypersil GOLD PREP C18 100 × 21.2 mm, 5 μM HPLC column (Thermo Fischer) connected to a guard column. Before injecting into the column, we dissolved the sample in TFA at approximately 50 mg per 500 μL TFA. We used a gradient of water with 0.1% TFA and acetonitrile with 0.1% TFA. We measured ultraviolet–visible (UV–vis) absorbance at 215 nm. We purified unlabeled Q11 (no ^13^C or ^15^N enrichment) and Q11 uniformly labeled with ^13^C and ^15^N at Q2, K3, and E9 to at least 95% purity on a Shimadzu Nexera HPLC system with a Waters XBridge C4 column. Again, we used water with 0.1% TFA and acetonitrile with 0.1% TFA as the mobile and stationary phases and monitored the peptide absorbance at 215 nm. We report HPLC chromatograms and mass spectrometry spectra in Figures S6 −S11.

### Fourier-Transform Infrared Spectroscopy

We prepared Q11 samples for Fourier-transform infrared spectroscopy (FTIR) measurements at 11 mg/mL peptide concentration in DI water and left them at 4 °C overnight to cure. Prior to FTIR measurement, we added 10× PBS, containing 1.37 M NaCl, 27 mM KCl, 100 mM Na_2_HPO_4_, and 18 mM KH_2_PO_4_ at pH 7.4, to obtain a final peptide concentration of 10 mg/mL (or 6.6 mM) in 1× PBS. We then spotted the peptide sample onto a Thermo Scientific Nicolet 6700 spectrometer with an attenuated total reflection (ATR) accessory after blanking with 1× PBS. Values are reported as an average from 128 scans. We calculated the β-index of the spectra using baseline correction, data smoothing, and Gaussian peak fitting in Wolfram Mathematica.

### Thioflavin T Fluorescence

We dissolved the Q11 peptide to a concentration of 1 mg/mL (or 0.66 mM), 0.08 mg/mL Thioflavin T (ThT), and 1× PBS before dispensing the solution to a black 96-well plate (Thermo Scientific Nunc). We measured the fluorescence intensity using a BioTek Synergy H4 Microplate Reader (excitation 450 nm, emission 482 nm, slit bandwidth 9 nm) over 48 h in triplicate, with the average of the samples reported in the main text.

### Molecular Modeling of β-Sheet Nanofibers

We built in-register parallel and in-register antiparallel β-sheet all-atom structural models using nanoscale molecular dynamics (NAMD) and visual molecular dynamics (VMD) software to estimate possible NMR-detectable distances in solid-state NMR experiments.^10−12^ To construct a Q11 monomer, we used the Molefacture feature in VMD using the known amino acid sequence. We then implemented custom Wolfram Mathematica code to form two β-sheets composed of 10 Q11 monomers each stacked with their hydrophobic faces pointing toward each other to form a hydrophobic core. We prepared the initial Q11 nanofiber models for four basic nanofiber configurations: parallel β-sheets with parallel stacking between sheets, parallel β-sheets with antiparallel stacking between sheets, antiparallel β-sheets with parallel stacking between sheets, and antiparallel β-sheets with antiparallel stacking between sheets. For each of the four configurations, we ran the following four steps of molecular dynamics simulations:

1. First, we randomized the initial side chain conformations while fixing the backbone carbon and nitrogen positions. We ran a minimization step of 10 ps, then raised the temperature from 0 to 300 K in increments of 10 K with 10 ps of production at each temperature. We ran an additional 10 ps of minimization after the temperature ramp.
2. To bring the Q11 monomers together to form β-sheets, we ran a 20-ps energy minimization step followed by a 10-ps production step. At this step, we removed the backbone nitrogen and carbon position constraints and introduced artificial dihedral angle, hydrogen bond, and hydrogen bond angle constraints into the simulations.
3. We then introduced artificial bonds between α carbons in the peptide backbones of the two stacked sheets to bring the two β-sheets together to form a nanofiber with a hydrophobic core. Again, we ran an energy minimization step of 10 ps followed by a temperature ramp to 300 K in increments of 10 K from 0 K with 10 ps of production at each temperature. We ran an additional 20 ps of minimization after the temperature ramp.
4. Lastly, we relaxed the spring constant from *k* = 1 to *k* = 0.1 for all constraints and ran the nanofiber structure for another 40 ps of production followed by 40 ps of minimization.

Four-layer molecular models were built following the same steps listed above, with an additional set of hydrogen bond and bond angle constraints to bring together all four β-sheets (Step 3).

### Solid-State NMR Measurements

We prepared Q11 peptides for NMR measurements at 10 mg/mL (or 6.6 mM) peptide concentration in water then left the samples at 4 °C overnight following established protocols for the self-assembly of Q11.^2,13−15^ We then diluted the solutions to 1 mg/mL peptide concentration using 1x PBS. To pack the samples into Bruker 3.2 mm NMR rotors (sample holders), we ultracentrifuged samples at 150,000 RCF and 4 °C for 30 min using a custom widget in Ultraclear tubes and a SW-41 swinging bucket rotor on a Beckman Optima XPN-100 Centrifuge. We used an 11.75 T magnet (500 MHz, ^1^H NMR frequency) with a Bruker Low-E ^1^H/^13^C/^15^N NMR probe to collect all NMR spectra. Prior to Q11 sample data collection, we calibrated the magnet with ^13^C chemical shift referencing to tetramethylsilane using an adamantane standard. All measurements were conducted at room temperature.

We collected ^1^H–^13^C CPMAS and 2D DARR measurements at a magic angle spinning speed of 10 kHz. We collected 2D ^13^C–^13^C spectra, using the DARR dipolar recoupling technique during mixing, at 50 ms mixing time to identify amino acid chemical shifts for each ^13^C uniformly labeled residue. For DARR recoupling, we applied continuous irradiation and power corresponding to 10 kHz nutation frequencies (and MAS spinning speed) during mixing time.^8^ At 50 ms mixing time, ^13^C–^13^C crosspeaks mostly correspond to ^13^C atoms within a single amino acid. We also collected 2D ^13^C–^13^C DARR spectra at 500 ms mixing time to observe additional crosspeaks that provide information on longer range couplings such as those between residues close in proximity (<6 Å).

We performed PITHIRDS-CT experiments at a MAS spin rate of 12.5 kHz. ^13^C–^13^C dipolar recoupling times were adjusted by adjusting the number of blocks of pulses (*k1*, *k2*, and *k3* defined by Tycko et al.) to collect signal intensities between 0 and 61.44 ms.^16^ We applied ^1^H decoupling at 100 kHz during recoupling periods and data acquisition.

We conducted ^15^N{^13^C} REDOR experiments at 10 kHz MAS with ^13^C and ^15^N π pulses set to 10 μs.^17^ We used the *cpredori* standard Bruker pulse program, which alternates scans to measure S and S0. We also used the tppm15 decoupling sequence, with proton decoupling power set to 100 kHz and ^13^C and ^15^N 10 μs π pulses. The contact time for cross-polarization was 1 ms with *ramp50100.100*. We used a recycle delay (d1) of 4s, acquisition time (AQ), of 10.24 ms, and dwell time (DW) of 5 μs.

To evaluate salt bridge formation between residues, we performed fsREDOR measurements at 10 kHz MAS. We applied frequency-selective Gaussian π pulses (400 μs) at the frequencies for the amine nitrogen of K3 (K3 Nζ) and the carboxylate carbon of E9 (E9 Cδ) as described by Jaroniec et al.^9^ We used the *swftppm* decoupling sequence with 1 ms contact time and the ramp10070.100 ramp for variable amplitude for hydrogen carbon α cross-polarization (HCα CP). The center frequency for the ^13^C selective pulse was at 181 ppm for the E9 Cδ, and the ^15^N selective pulse was at 33.4 ppm for K3 Nζ. The selective Gaussian pulses for peak inversion in fsREDOR were optimized using the *cpsel_inv* standard Bruker pulse program, following Li et al.^18^ We set the Gaussian pulse lengths for both ^13^C and ^15^N to 400 μs. We used a recycle delay (d1) of 4s, acquisition time (AQ), of 12.29 ms, and dwell time (DW) of 12 μs.

REDOR and fsREDOR data are reported as the difference between measured ^13^C peak intensity with ^15^N pulses (S) scaled to the peak intensity measured from the same sequence of NMR pulses without the ^15^N pulses (S_0_). This scheme isolates the heteronuclear dipolar coupling from other effects that can also affect NMR signal intensity.^9^ To obtain the peak intensities for PITHIRDS-CT, REDOR, and fsREDOR, we used Wolfram Mathematica’s NonlinearModelFit function for nonlinear Gaussian peak fitting. The PITHIRDS-CT curve also accounts for natural abundance correction on backbone carbonyls with chemical shifts that overlap with the largest peak at 171 ppm. We included error bars for all dipolar recoupling experiments based on a 95% confidence interval. We reported NMR spectra collected with 12 h of signal averaging.

### Nuclear Spin Simulations of Dipolar Recoupling NMR Experiments

We used SpinEvolution NMR simulation software to simulate ^13^C PITHIRDS-CT and ^15^N{^13^C} REDOR measurements on linear eight-spin systems modeled as a linear array of eight spins with constant distances set at 4 and 5 Å. We set simulation parameters to match experimental conditions.^9,16^ We simulated PITHIRDS-CT experiments with eight ^13^C atoms and REDOR simulations with four ^13^C and four ^15^N atoms. We used anisotropic parameters (δ_aniso_=–75 ppm, η_Ω_ = 0.75, α_Ω_ = 0°, β_Ω_ = 0°, γ_Ω_ = 0°) to account for chemical shift anisotropy effects in simulations of PITHIRDS-CT. Figure S5 compares the differences between spin simulations generated with two spins vs eight spins.

## Results

### Q11 peptide nanofibers exhibited ThT fluorescence, transmission electron microscopy (TEM), and attenuated total reflectance–Fourier transform infrared spectroscopy (ATR-FTIR) measurements consistent with prior reports of Q11

**Figure 1:**
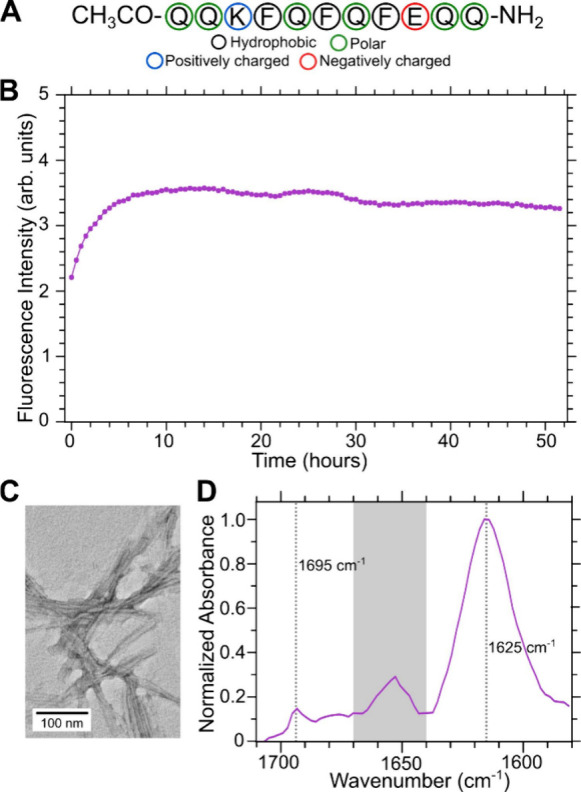
Q11 peptide sequence and results from biophysical measurements. (A) Q11 amino acid sequence, with colored circles identifying hydrophobic, polar, positively charged, and negatively charged residues. (B) ThT fluorescence measurement of Q11 (1 mg/mL in 1× PBS). (C) Transmission electron micrograph of Q11 nanofibers (1 mg/mL in 1× PBS). (D) ATR-FTIR spectrum of Q11 (10 mg/mL in 1× PBS). Gray dashed lines highlight peaks at 1695 and 1625 cm^–1^, indicating the presence of antiparallel β-sheets. The gray highlighted region between 1640 and 1670 cm^–1^ can be attributed to random coil, α-helical structures, or contributions from the amide I bond stretching from the amine group in the side chain of glutamine.

We anticipated that the Q11 amino acid sequence would promote antiparallel β-sheet formation due to the alternating hydrophobic F and polar Q amino acids along its hydrophobic face and the K and E charged amino acids positioned at opposite ends of the sequence (Figure 1A). The termini are acetylated at the N-terminus and amidated at the C-terminus to avoid interactions with charged termini. ThT fluorescence of Q11 at 1 mg/mL (or 0.66 mM) showed an increase in average fluorescence intensity over time, confirming the assembly of Q11 peptides into β-sheets. The ThT curves indicate that assembly occurs immediately after dissolution and plateaus at around 10 h (Figure 1B). ThT replicate curves are shown in Figure S1. Previous work also demonstrated an increase in ThT fluorescence intensity over time as the ThT molecule binds to Q11 β-sheets.^19−21^ We confirmed the presence of Q11 nanofibers from TEM images of Q11 prepared at 1 mg/mL in 1x PBS. These nanofibers are morphologically similar to the nanofibers reported in existing Q11 literature (Figure 1C).^1^ We also observed interfibrillar associations via TEM.

For our ATR-FTIR measurements, we prepared Q11 at a higher peptide concentration of 10 mg/mL (or 6.6 mM) in 1× PBS to obtain better surface coverage of the peptide nanofibers on the ATR module. We identified Q11 β-sheet formation based on the FTIR peak at 1625 cm^–1^ (Figure 1D).^22^ The peak at 1695 cm^–1^ was previously attributed to the presence of antiparallel β-sheets by Collier et al.^1^ If entirely parallel β-sheets were present in the sample, a single peak at around 1620 cm^–1^ would be expected with no peak at 1695 cm^–1^.^22−26^ FTIR peaks between 1640 to 1670 cm^–1^ were also present. Peaks within this range have been attributed to the presence of random coil and α-helices, but the peaks can also contain contributions from the amide bond stretching from the amine group in the side chain of glutamine.^1,27^ We also conducted ATR-FTIR on a Q11 sample prepared at 40 mg/mL (or 26 mM) in 1:1 H_2_O:D_2_O, following the sample preparation protocol used by Collier et al.^1^ Our results are consistent with previously reported FTIR measurements (Figure S2). Biophysical measurements from ATR-FTIR and ThT showed that the nanofibers captured from TEM images are β-sheet rich as expected.

We conducted additional FTIR analysis using a quantitative approach described by Celej et al. to empirically distinguish between parallel and antiparallel β-sheets. We calculated a “β-sheet organizational index”, or “β-index”, which has been used for detailed spectral analysis of Alzheimer’s amyloid-β peptide aggregates.^28,29^ The β-index is the ratio of the intensity of the peak between 1693 and 1697 cm^–1^ to the intensity of the peak between 1624 and 1632 cm^–1^.^28,29^ Celej et al. and Hubin et al. used a β-index value of 0.1 to classify parallel vs antiparallel β-sheets. β-index values below 0.1 were assigned to parallel β-sheets while values above 0.1 were assigned to antiparallel β-sheets.^28,29^ Our β-index analysis of the Q11 spectra gave a β-index value of 0.082 which would be marginally classified as parallel β-sheets according to this quantitative and empirical approach.

### 2D ^13^C–^13^C NMR and fsREDOR measurements showed spatial proximity between K3 and E9 residues

**Figure 2:**
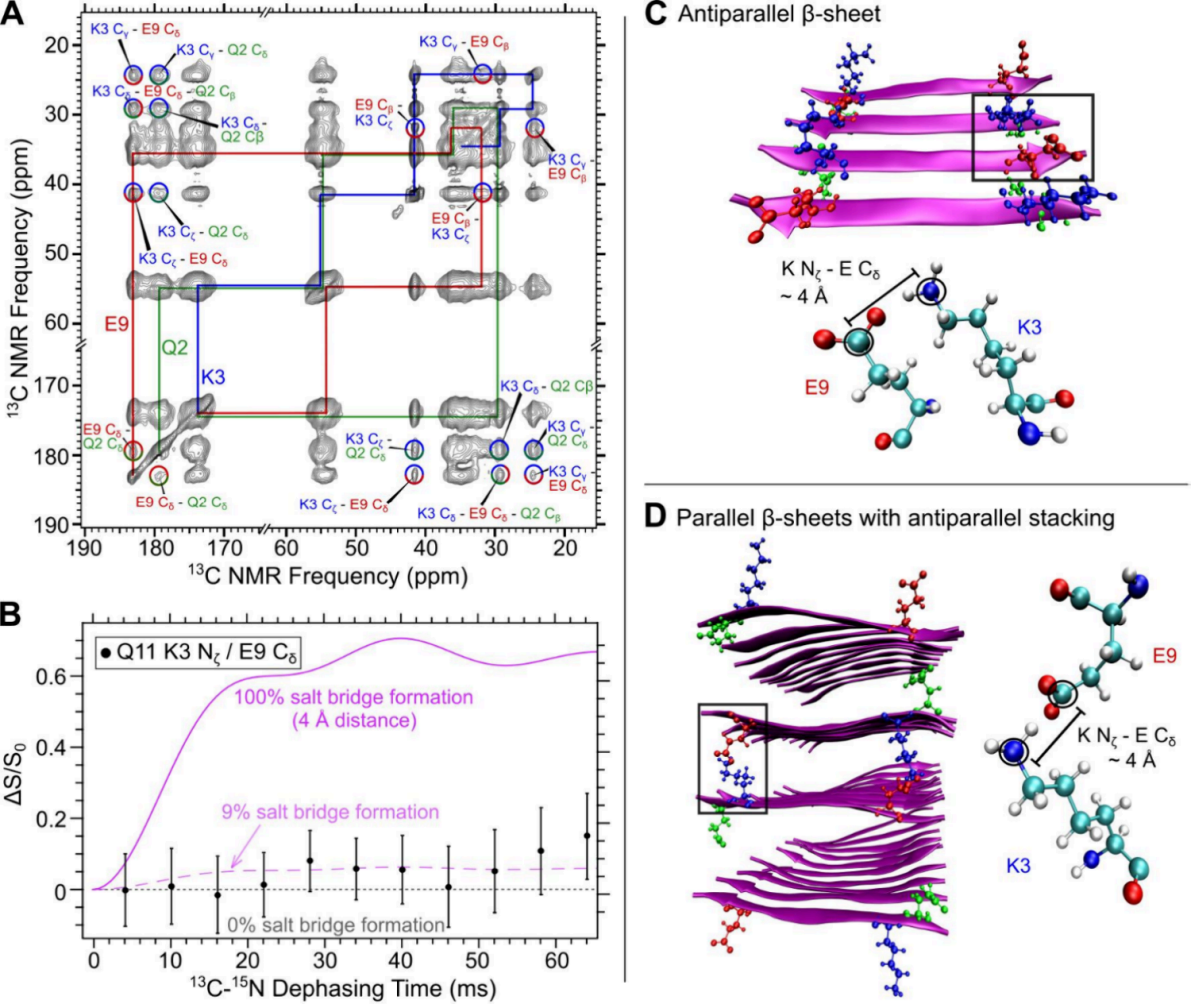
2D NMR measurements on Q11 uniformly ^13^C and ^15^N labeled at residues Q2, K3, and E9 (Sample 1) and molecular models highlighting labeled sites. (A) ^13^C–^13^C DARR spectra collected at 500 ms mixing time. Blue, red, and green colored lines indicate spectral assignments based on 50 ms mixing time DARR measurements for lysine, glutamine, and glutamic acid, respectively (see Figures S4 and S5). Colored circles with two or three colors identify off-diagonal crosspeaks corresponding to atoms on different amino acids. (B) ^15^N{^13^C} fsREDOR measurements show proximity between K3 Nζ and E9 Cδ atoms. A dashed pink line showing a best-fit approximation of 9% salt bridge formation between K3 and E9. An fsREDOR simulation curve at 4 Å is also shown in pink. (C) Molecular model of Q11 antiparallel β-sheets. (D) Molecular model of Q11 parallel β-sheets stacked antiparallel between β-sheets. The approximate distance between K3 Nζ and E9 Cδ in both molecular models is 4 Å.

2D DARR provides information on distance-dependent spin–spin interactions between isotopically labeled sites. The presence of off-diagonal “crosspeaks” can report on ^13^C–^13^C couplings between residues that are within the NMR-detectable distance of 6 Å. For parallel β-sheets with parallel stacking, we would only expect to see crosspeaks between Q2 and K3 labeled sites. Crosspeaks between K3 and E9 residues can indicate antiparallel stacking between parallel β-sheets or can also be due to the antiparallel nearest neighbors. Figure 2A shows a ^13^C–^13^C correlation spectrum from DARR on a Q11 sample enriched with ^13^C and ^15^N at residues Q2, K3, and E9 (Sample 1). We identified off-diagonal crosspeaks between K3 and E9, as well as Q2 and K3. We also observed some crosspeaks between Q2 and E9, but they are difficult to distinguish because of the overlapping NMR peaks from K3. We show ^13^C peak assignments from 50 ms mixing time DARR in Figure S3 and an overlay of 50 ms mixing time and 500 ms mixing time DARR with 1D slices in Figure S4.

We also conducted ^15^N{^13^C} fsREDOR on Sample 1 to probe the spatial proximity between the labeled side chain amine nitrogen of K3 (Nζ) and the side chain carboxylate carbon of E9 (Cγ). This experiment is sensitive to distance-dependent dipolar coupling effects between heteronuclear atoms, in this case, between ^15^N and ^13^C. The frequency-selective pulses in fsREDOR isolate pairs of selected ^13^C and ^15^N atoms by eliminating the homonuclear dipolar coupling interactions of nearby ^13^C and ^15^N atoms.^30^ Side chain carboxylate ^13^C and ^15^N atoms have distinct NMR frequencies from other labeled sites which make them easier to isolate from other frequencies. Figure 2B shows a fsREDOR effect (Δ*S*/*S*_0_) between the ^13^C and ^15^N labeled sites. Figure 2B also shows a fsREDOR spin simulated curve at 4 Å between labeled sites, which would indicate 100% of all labeled K3 Nζ and E9 Cδ sites are within the distance for salt bridging to occur. We conducted a best-fit approximation using the method of least-squares and determined that the data are approximately 9% of the spin simulation curve at 4 Å and 91% of a flat line at 0 at all recoupling times (no salt bridging). About 9% of the K3 Nζ and E9 Cδ spins are coupled and within the 4 Å distance for salt bridging to occur. The spatial proximity between K3 and E9 can be explained by two possible conformations: antiparallel β-sheets (Figure 2C) and antiparallel stacking between parallel β-sheets (Figure 2D). We are unable to differentiate between the two molecular models from 2D DARR and fsREDOR alone.

### Q11 peptides predominantly self-assembled into parallel β-sheets according to dipolar recoupling solid-state NMR measurements

**Figure 3:**
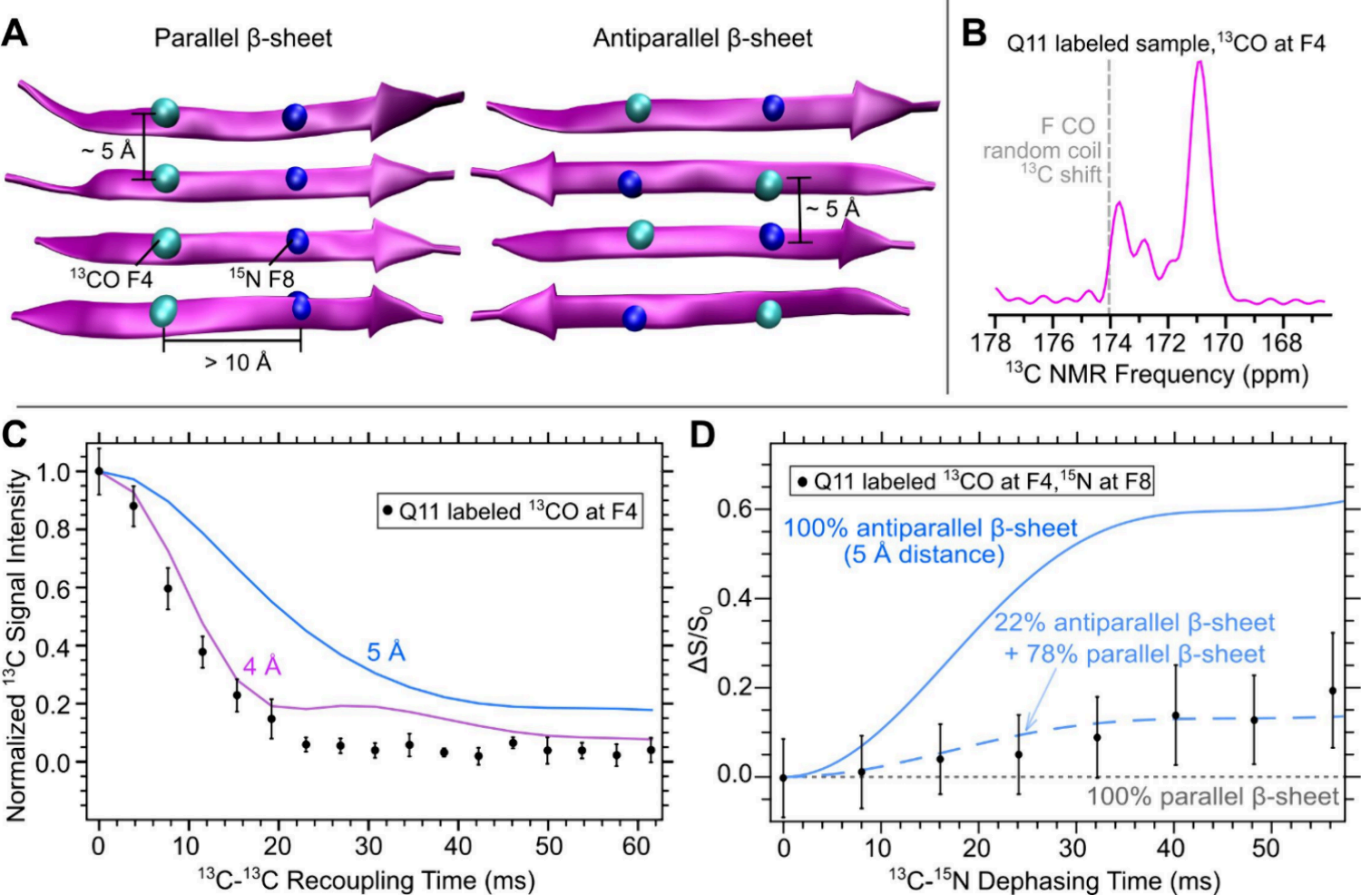
Dipolar recoupling NMR measurements on Q11 labeled with ^13^CO at F4 and ^15^N at F8 (Sample 2) suggest a heterogeneous structure with mostly parallel and some antiparallel β-sheet content. (A) Molecular models of idealized parallel and antiparallel β-sheets. Labeled carbon and nitrogen atoms are colored in cyan and blue, respectively. Approximate distances between β-strands are shown. (B) Carbonyl region of a 1D ^13^C NMR spectrum of Sample 2. Gray dotted line denotes the expected random coil chemical shift for F CO. (C) ^13^C PITHIRDS-CT measurement on Sample 2. (D) ^15^N{^13^C} REDOR measurement on Sample 2. Both PITHIRDS-CT and REDOR data were obtained from analysis of the largest peak at ∼171 ppm. Error bars represent a 95% confidence interval from nonlinear peak fitting to a Gaussian distribution. Simulated curves for dipolar recoupling NMR measurements on linear β-sheets are also shown in pink and blue at distances of 4 and 5 Å, respectively. The blue dashed line in (D) shows the best-fit curve of approximately 22% antiparallel and 78% parallel β-sheets.

To probe whether Q11 adopted parallel or antiparallel β-sheet configurations, we conducted ^13^C PITHIRDS-CT and ^15^N{^13^C} REDOR measurements on a Q11 peptide sample selectively labeled with ^13^CO on F4 and^15^ N on F8 (Sample 2). PITHIRDS-CT measures homonuclear ^13^C–^13^C dipolar couplings.^16^ Unlike fsREDOR, REDOR measures heteronuclear ^13^C–^15^N dipolar couplings throughout the sample. We used two simple molecular models of a parallel β-sheet and an antiparallel β-sheet to select the ^13^C and ^15^N labeled sites and serve as a point of reference to discuss our results (Figure 3A).^16^ To distinguish between parallel and antiparallel β-sheets, we used spin simulations on linear arrays of ^13^C and ^15^N atoms. Within parallel β-sheets, ^13^CO F4 sites on each β-strand are ∼5 Å apart, close enough to have strong dipolar couplings, and show a significant PITHIRDS-CT decay. Antiparallel β-sheets are organized such that the ^13^CO F4 and ^15^N F8 sites are also within distances of 5 Å to show a REDOR effect. Conversely, we would expect no measurable decay in PITHIRDS-CT experiment for antiparallel β-sheets and little REDOR effect for parallel β-sheets since dipolar couplings are weaker between atoms that are greater than 10 Å apart, corresponding to little dipolar recoupling. However, ^13^C NMR measurement by ^1^H–^13^C CPMAS revealed complexity that affects our interpretation of data: Figure 3B shows multiple NMR peaks in the carbonyl region demonstrating that Q11 adopts multiple nanofiber structures; the labeled ^13^CO F4 carbon atoms experience multiple local environments with distinct chemical shifts. To probe the most abundant structure in the sample, both PITHIRDS-CT and REDOR analyses were conducted on the largest peak at 171 ppm.^29^

The measured PITHIRDS-CT decay from Sample 2 shows a nearly 100% decrease in ^13^C signal intensity within 20 ms of ^13^C–^13^C recoupling time (Figure 3C). This behavior indicates the parallel organization of Q11 β-strands into β-sheets. Compared to the spin simulations, our PITHIRDS-CT data reveal a discrepancy between the measured decay and the decay predicted by simulations. The data seem to correspond to ^13^C–^13^C distances of 4 Å rather than 5 Å. While systematic deviations between measured and simulated PITHIRDS-CT curves have been reported before, data usually underpredict decays compared to simulations.^16,31,32^Figure 3D shows a detectable REDOR effect (Δ*S*/*S*_0_) that approaches 0.2 at long recoupling time. We again conducted a best-fit approximation between the REDOR data and nuclear spin simulations on a linear array of alternating ^13^C and ^15^N atoms spaced at a constant distance of 4 or 5 Å apart. Data that closely follow the spin simulation curve at 5 Å would indicate 100% antiparallel β-sheets. A parallel β-sheet would produce no REDOR effect, or a flat line at 0 for all recoupling times. Our best-fit approximation showed that a curve that is 22% antiparallel and 78% parallel β-sheets is consistent with the REDOR data. However, since we do not know the full nature of the structural heterogeneity, it is difficult to assess the accuracy of this quantification.

We cannot determine the precise distribution of ^13^C and ^15^N distances and provide detailed explanations of discrepancies between simulations and our REDOR data because we have a structurally heterogeneous sample. The relatively low REDOR and fsREDOR effects could be explained in two ways: (1) a homogeneous structure with long distances between the labeled sites or (2) a heterogeneous structure with short and long distances between the labeled sites. The second reason is the most likely scenario based on our other dipolar recoupling measurements. In both Figures 2B and 3D, we obtained REDOR curves that reached approximately 0.2 at long recoupling times. It is unclear whether the REDOR curves plateau or are still building up past 60 ms because of the heterogeneity. We also compared two-spin REDOR simulations with eight-spin simulations in Figure S5. The eight-spin simulations go up to about Δ*S*/*S*_0_ = ∼ 0.6, while the two-spin simulations go up to Δ*S*/*S*_0_ = ∼ 1. Multispin effects can bring down the dephasing, and REDOR effects can be reduced with homonuclear ^13^C–^13^C couplings in the sample (and in our simulations). While our simple molecular models of parallel and antiparallel β-sheets guide the discussion of the experimental results, the models do not fully capture the structural heterogeneity or distribution of structures in the sample.

**Figure 4:**
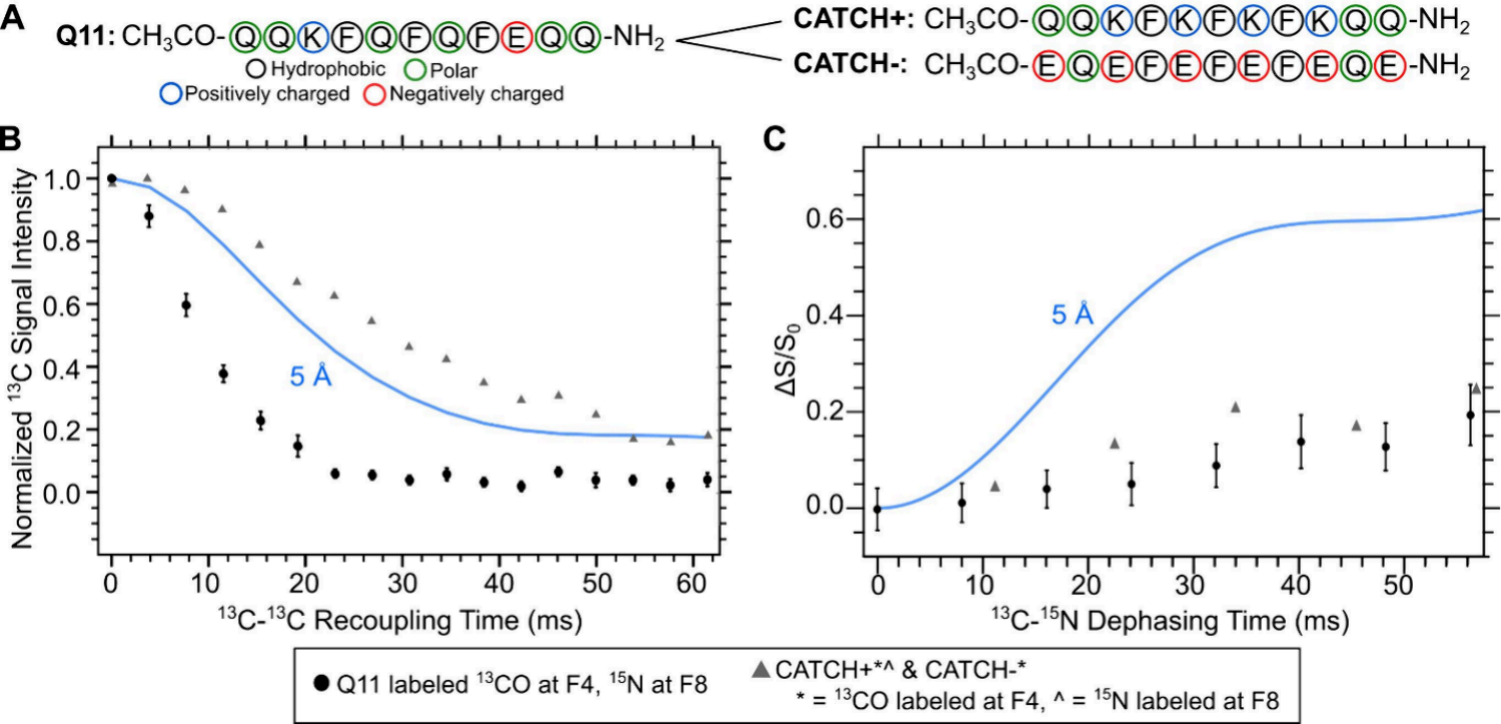
Comparison of Q11 and coassembled CATCH+/CATCH- dipolar recoupling measurements. (A) Q11, CATCH+, and CATCH– amino acid sequences. Overlay of (B) ^13^C PITHIRDS-CT and (C) ^15^N{^13^C} REDOR measurements on labeled Q11 and CATCH+/CATCH– nanofibers. A spin simulation curve for PITHIRDS-CT and an fsREDOR spin simulation curve at 5 Å are also shown for reference.

Structural studies on the CATCH “co-assembling” peptide system, which was inspired by the Q11 sequence design, showed that the resulting CATCH nanofibers are also structurally heterogeneous.^33^ Seroski et al. designed charge complementary peptide variants of Q11, CATCH+ (Ac-QQKFKFKFKQQ-Am), and CATCH– (Ac-EQEFEFEFEQE-Am), which only assemble into β-sheet nanofibers when combined (or “co-assembled”) and do not self-assemble when prepared separately.^34^ Structural analysis using NMR measurements and computational approaches on CATCH nanofibers revealed a mixture of in-register parallel, in-register antiparallel, out-of-register β-sheets, and self-association of CATCH+ and CATCH– peptides, with no preference for a single structure.^33^Figure 4 shows an overlay of the dipolar recoupling measurements from Q11 and the CATCH peptides. Compared to Q11 peptide nanofibers, the PITHIRDS decay of the coassembled CATCH peptide nanofibers has a weaker decay. Both samples have REDOR curves that are relatively low compared to the spin simulation.

## Discussion

We sought to evaluate whether the heuristics such as hydrophobic and polar amino acid patterning and strategic placement of oppositely charged K and E side chains can control β-strand arrangements within Q11 β-sheets. Previous work using commonly used biophysical characterization techniques corroborate expectations for Q11 peptide self-assembly: the peptide assembled into fibrils visible by electron microscopy and atomic force microscopy, circular dichroism showed β-strands, and FTIR showed the presence of peaks expected for antiparallel β-sheets. We also ran solid-state NMR to provide molecular-level detail into the structure. Our 2D DARR and fsREDOR results suggested that K3 and E9 residues are within spatial proximity. From the biophysical measurements and 2D NMR results alone, we would have concluded that Q11 nanofibers formed antiparallel β-sheets. However, further dipolar recoupling NMR experiments revealed primarily parallel β-sheet formation and structural heterogeneity within Q11 nanofibers. The standard set of biophysical characterization techniques and analysis can be misleading in the presence of structural disorder. From these results, we revealed that the heuristics-based amino acid sequence design of Q11 is not necessarily precise in controlling β-sheet structures.

Our biophysical measurements were consistent with previous reports of Q11, but further FTIR analysis revealed potential structural heterogeneity. We confirmed the presence of nanofibers from TEM and detected an increase in fluorescence intensity consistent with β-sheet formation from ThT fluorescence.^19,20^ We showed the presence of β-sheets and a peak at 1695 cm^–1^ from ATR-FTIR, consistent with previously reported results by Collier et al.^1^ The peak at 1695 cm^–1^ can be misinterpreted to mean that the entire sample consists of antiparallel β-sheets. In structural studies of the amyloid-β peptide, it took decades of FTIR and solid-state NMR research to understand that smaller insoluble aggregates, often referred to as “oligomers”, tend to be transient antiparallel β-sheet structures.^24,35^ Celej et al. use an empirical threshold for a quantitative β-index term to analyze the FTIR data and differentiate between parallel vs antiparallel β-sheets. Our FTIR data on Q11 revealed a β-index value of 0.082 which is marginally more consistent with parallel β-sheets than antiparallel β-sheets in amyloid-β FTIR literature. The presence of a peak at approximately 1690 cm^–1^ does not necessarily mean that all the peptides in the sample are arranged into antiparallel β-sheets. When analyzed in this quantitative manner, the FTIR data and NMR data reporting structural heterogeneity of Q11 are harmonious.^24^

Our solid-state NMR measurements on isotopically enriched Q11 samples revealed primarily parallel β-sheets and some antiparallel β-sheet content. ^13^C–^13^C DARR measurements show off-diagonal crosspeaks between K3 and E9, indicating these residues are within 6 Å (Figure 2A). fsREDOR measurements also showed proximity between the side chain carboxylate carbon of E9 and amine nitrogen of K3 (Figure 2B), and we estimated about 9% of K3 and E9 residue pairs form salt bridges from best-fit approximations to simulated fsREDOR curves. The proximity provides some evidence for salt bridge formation; however, the strength of the coupling is too weak to be descriptive of the whole sample. The DARR and fsREDOR measurements can be rationalized by either antiparallel stacking between β-sheets or antiparallel β-sheet formation. A strong PITHIRDS-CT signal decay indicates that β-strands adopted mostly parallel β-sheet conformations (Figure 3C). The measured ^15^N{^13^C} REDOR effect reveals that antiparallel β-strands also sit next to parallel β-strands producing a structurally heterogeneous β-sheet (Figure 3D).^22^ A best-fit approximation showed a curve that is 22% antiparallel and 78% parallel β-sheets is consistent with the REDOR data. However, the precision of this best fit is unknown because we do not fully understand the nature of structural heterogeneity. Collectively, the NMR results show that patterning of hydrophobic and polar amino acids promotes β-sheet formation, but the placement of charged amino acids on opposite ends of the amino acid sequence does not bias toward the intended antiparallel β-sheet structure.

Our structural measurements on Q11 and existing literature on β-sheet peptide structure suggest that we do not have a systematic way of predicting monomorphism or polymorphism in a β-sheet peptide assembly. Previous structural characterization on MAX1 (VKVKVKVKV^D^PPTKVKVKVKV) showed a highly ordered and monomorphic peptide assembly.^36^ On the other hand, structural measurements on RADA16-I (Ac-RADARADARADARADA-Am) showed a major structure that is highly ordered but has some evidence of a minor structure.^37^ Compared to MAX1 and RADA16-I, Q11 peptide nanofibers are more polymorphic, although we cannot make a comprehensive assessment because samples were prepared at different assembly conditions depending on their use as a biomaterial. Considering the broader story of peptide self-assembly to include naturally occurring peptides (such as the Alzheimer’s Aβ peptide), the assembly conditions influence the degree of polymorphism in the final assembled structure. However, our knowledge of the relationship between assembly conditions and structural homogeneity is limited, especially when looking at designer peptides. While it is possible to explore experimental conditions to achieve a monomorphic structure, we currently do not have evidence that such a set of conditions exists for Q11. In this work, we only prepared samples according to the conventional use of Q11 as a biomaterial. Impurities can also affect the assembly process and contribute to structural heterogeneity. However, we cannot conclude whether purifying Q11 to 100% purity would make the peptide nanofibers less polymorphic. We think that computational peptide sequence design is a better approach to reduce the likelihood of polymorphism by simulating peptide assembly in typical experimental conditions. Our ongoing work on computationally-designed peptides presents an opportunity for better-controlled β-sheet peptide assembly designs.^38,39^

Compared to the CATCH coassembled system, Q11 shows a stronger preference for parallel β-sheets because of the stronger PITHIRDS-CT decay and a weaker REDOR effect (Figure 4). NMR measurements have shown that charge patterning within peptides can resist self-assembly and promote coassembly, but this rule is not effective at promoting structural order.^21,33,40^ Electrostatic interactions between lysine and glutamic acid were not enough to bias Q11 peptides to self-assemble into antiparallel β-sheets. The charge modifications of CATCH+ and CATCH- were also unable to produce a higher-ordered, electrostatically controlled assembly.^38^ Structural characterization on another peptide coassembling system, the King-Webb peptides (KW+: KKFEWEFEKK and KW-: EEFKWKFKEE), also shows structural disorder but the overall charge of each complementary peptide was closer to neutral.^40^ We suggest that a degree of structural disorder detected in the CATCH system may be “inherited” from the Q11 amino acid sequence rather than simply a feature of coassembly. We note, however, that not all peptide coassemblies are disordered. Another type of peptide coassembly which occurs from a racemic mixture of the same peptide forms highly ordered crystal structures that form rippled β-sheets rather than pleated β-sheets.^41^ Further research into the molecular structures formed by peptides can provide more information about the peptide coassembly process.

Our NMR investigation illustrates the complexity involved with analyzing peptide nanofibers with structural heterogeneity. Conventional NMR papers on peptide assemblies interpret results with the lens of homogeneity. If we had only evaluated the structure based on the amino acid sequence and the FTIR,, 2D DARR, and fsREDOR measurements alone, we would have assumed that Q11 peptide form antiparallel β-sheets. However, our dipolar recoupling measurements tell us a different story. The combination of PITHIRDS-CT and REDOR data indicates a combination of both parallel and antiparallel β-sheets. We think that our simple representation of antiparallel and parallel β-sheet molecular models, which assume perfect β-strand registry within the β-sheets, is the best possible approach to describe a sample with multiple possible structures. We do not have sufficient information to infer anything about the organization within the less abundant structures. Other biophysical techniques, such as AFM or cryo-EM, can provide additional insight into structure, although the polymorphic structure of Q11 can make structural data interpretation especially challenging. Nonetheless, our structural analysis contributes important information on the incomplete relationship between amino acid sequence design and assembled structure. In the bigger picture, we wish to investigate the overall problem of designing peptide assemblies to adopt controlled structures.

Overall, our NMR measurements gave us detailed molecular insight into the different structural features present within Q11 self-assembled nanofibers, demonstrating that the heuristics-based design for β-sheets is not always effective in designing for a specific structural arrangement. We cannot propose a complete structural model for Q11 because of the disorder in the Q11 nanofibers. We presently have no mechanism for predicting whether peptides assemble to form ordered or disordered structures. A central scientific issue here is the dependence of biological effects (e.g., immune responses) on molecular structure. Without precise control of structure, it may be impossible for us to pin down structure–function relationships. However, recent efforts have been placed into the development of computational peptide design algorithms to selectively form parallel and antiparallel β-sheets.^38,39^

## Conclusions

Our structural measurements on Q11 nanofibers show that although the heuristics-based design works to an extent in forming β-sheets, the β-sheet structures produced by Q11 peptides are not homogeneous or ordered. Unlike other self-assembling peptides RADA16-I and MAX1, Q11 peptides form a more polymorphic fibril structure. Q11 peptides primarily forms parallel β-sheets, with evidence for some antiparallel β-strand nearest neighbors and possible antiparallel stacking from NMR measurements. We do not propose a complete structural model because of the structural complexity that comes with heterogeneous samples. The impact of structural heterogeneity on the performance of peptide-based biomaterials in biomedical applications remains to be determined. Nonetheless, the rational design of amino acid sequences for specific therapeutic applications can be improved with molecular-level insight into the structural features possible within the assembled peptide nanofibers.

## Supporting Information Available

ThT replicate curves of Q11 (Figure S1); FTIR of Q11 prepared at 40 mg/mL in 1:1 H_2_O:D_2_O (Figure S2); ^13^C–^13^C DARR spectrum of Q11 uniformly ^13^C and ^15^N labeled at residues Q2, K3, and E9 collected at 50 ms mixing time (Figure S3); overlay of ^13^C–^13^C DARR spectra collected at 50 and 500 ms mixing times, with 1D slices (Figure S4); REDOR spin simulation curves using 2 spins vs 8 spins at 4 and 5 Å distances (Figure S5); HPLC chromatogram of Q11 (Figure S6); ESI-MS spectrum of Q11 (Figure S7); LC-MS spectrum of Q11 labeled ^13^CO at F4, ^15^N at F8 (Figure S8); MALDI-MS spectrum of Q11 labeled ^13^CO at F4, ^15^N at F8 (Figure S9); HPLC chromatogram of Q11 uniformly ^13^C and ^15^N labeled at Q2, K3, E9 (Figure S10); ESI-MS spectrum of Q11 uniformly ^13^C and ^15^N labeled at Q2, K3, E9 (Figure S11) (PDF)

## Abbreviations

NMR: nuclear magnetic resonance
CPMAS: cross-polarization magic angle spinning
REDOR: rotational-echo, double-resonance
DARR: dipolar-assisted rotational resonance
TEM: transmission electron microscopy
ThT: thioflavin T
ATR-FTIR: attenuated total reflectance–Fourier transform infrared spectroscopy
