# Supplementary material for: Parallel β-Sheet Structure and Structural Heterogeneity Detected within Q11 Self-Assembling Peptide Nanofibers
Source: J Phys Chem B. 2024 May 24;128(22):5387–96. doi: 10.1021/acs.jpcb.4c00825 (PMC11163420; doi:10.1021/acs.jpcb.4c00825)
Supplement: Supplementary file 1 — jp4c00825_si_001.pdf [file jp4c00825_si_001.pdf]

Supporting Information

Parallel β-sheet Structure and Structural Heterogeneity Detected within Q11 Self-Assembling Peptide Nanofibers

*Alicia S. Robang[1], Kong M. Wong[1], Johannes Leisen[2], Renjie Liu[3], Walker L. Radford[2], Tarunya Rao Sudarshan[1], Gregory A. Hudalla[3], Anant K. Paravastu[1,4]**

1. School of Chemical and Biomolecular Engineering, Georgia Institute of Technology, Atlanta, GA 30332, USA
2. School of Chemistry & Biochemistry, Georgia Institute of Technology, Atlanta, GA 30332, USA
3. J. Crayton Pruitt Family Department of Biomedical Engineering, University of Florida, Gainesville, FL 32611, USA
4. Parker H. Petit Institute for Bioengineering and Biosciences, Georgia Institute of Technology, Atlanta, GA 30332, USA

*Correspondence should be addressed to A.K.P. (e-mail: anant.paravastu@chbe.gatech.edu)

KEYWORDS

Peptides and proteins, self-assembling peptide, nanofibers, nuclear magnetic resonance

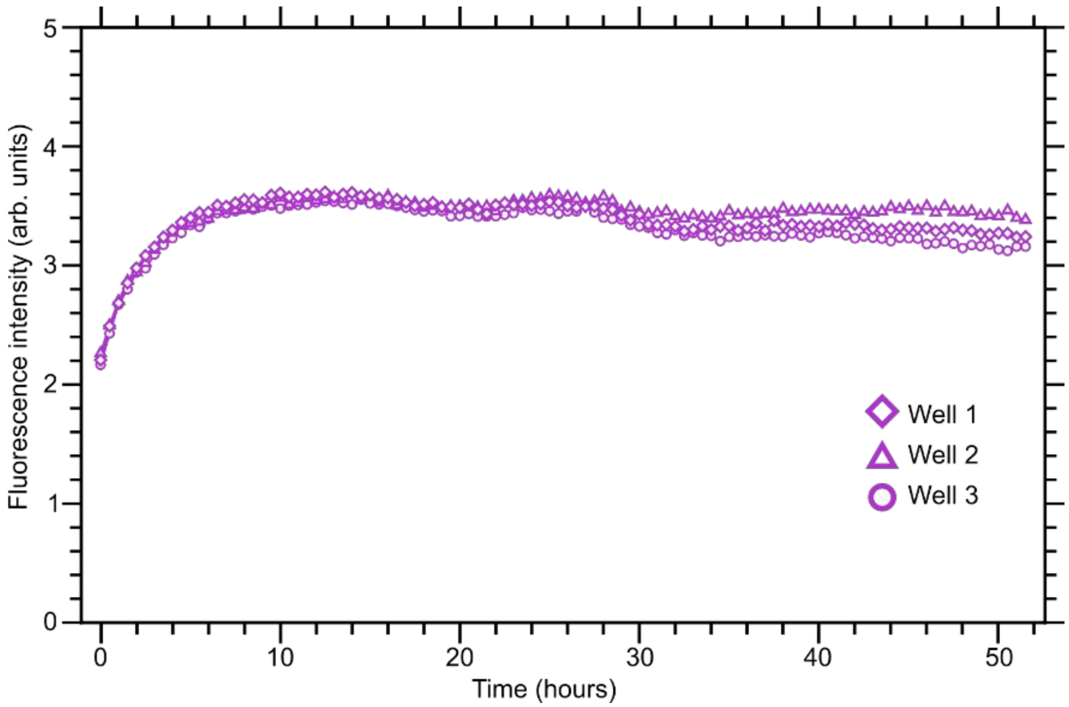

Figure S1. Thioflavin T fluorescence curves of Q11 prepared at 1 mg/ml in 1x PBS.

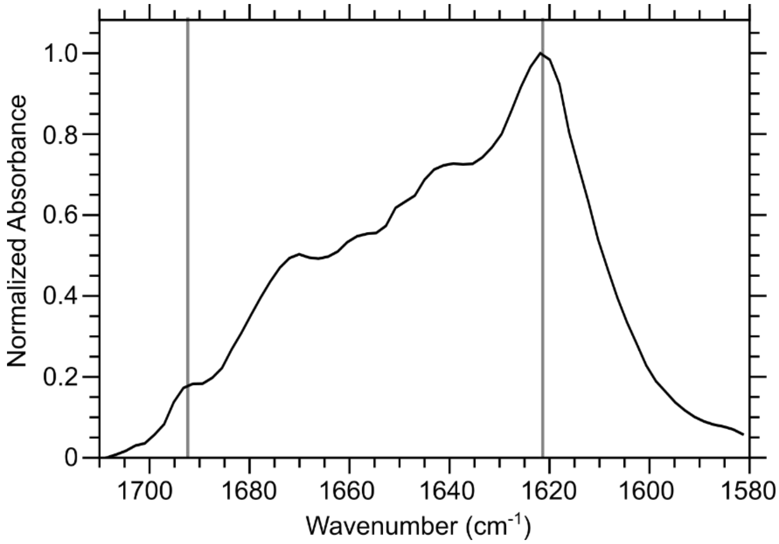

Figure S2. FTIR of Q11 prepared at 40 mg/ml in 1:1 H_2O:D_2O. Peaks observed at ~ 1625 cm^{-1} and ~ 1695 cm^{-1} indicate presence of antiparallel β-sheets. Peaks between 1640 and 1670 cm^{-1} can be attributed to other structures such as random coil, α-helices, or the amide bond stretching from the side chain of glutamine.

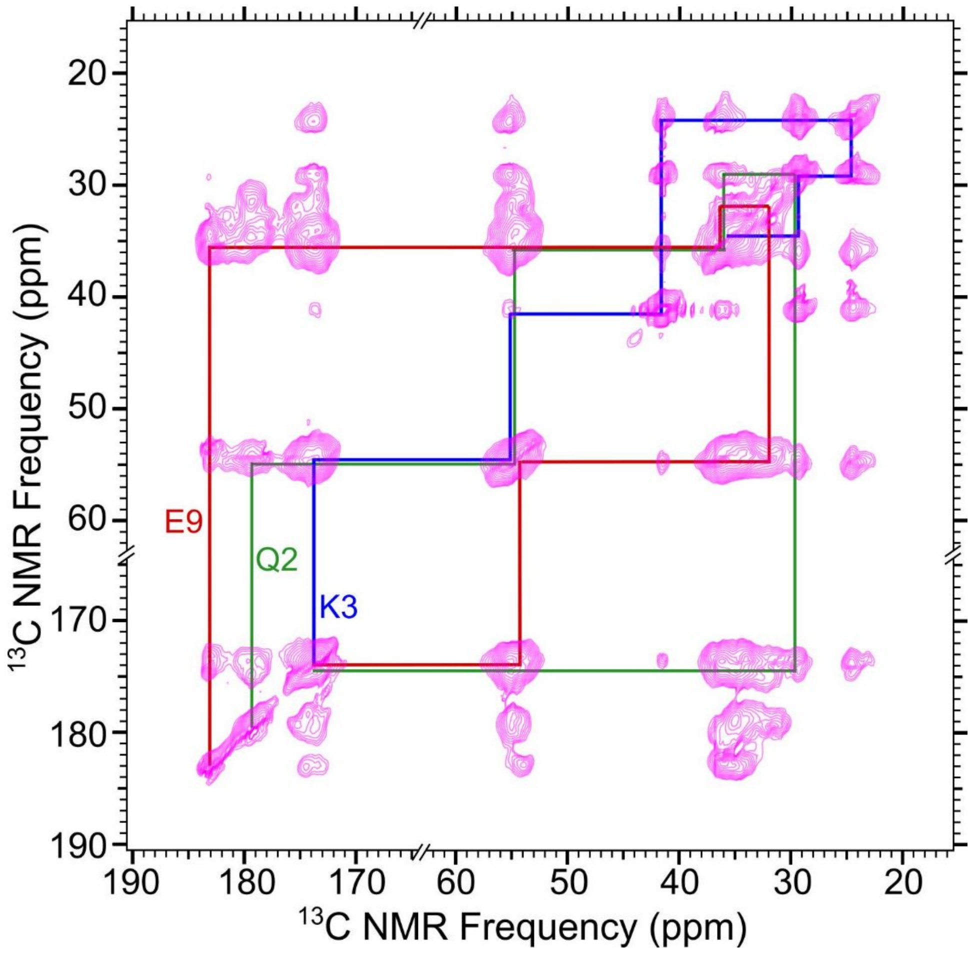

Figure S3. 2D ^{13}C-^{13}C DARR spectrum of Q11 uniformly ^{13}C and ^{15}N labeled at residues Q2, K3, and E9 collected at 50 ms mixing time ("short mixing time" DARR). Blue, green, and red solid-colored lines indicate spectral assignments made using the known amino acid chemical shifts for carbons in β-sheet secondary structure.

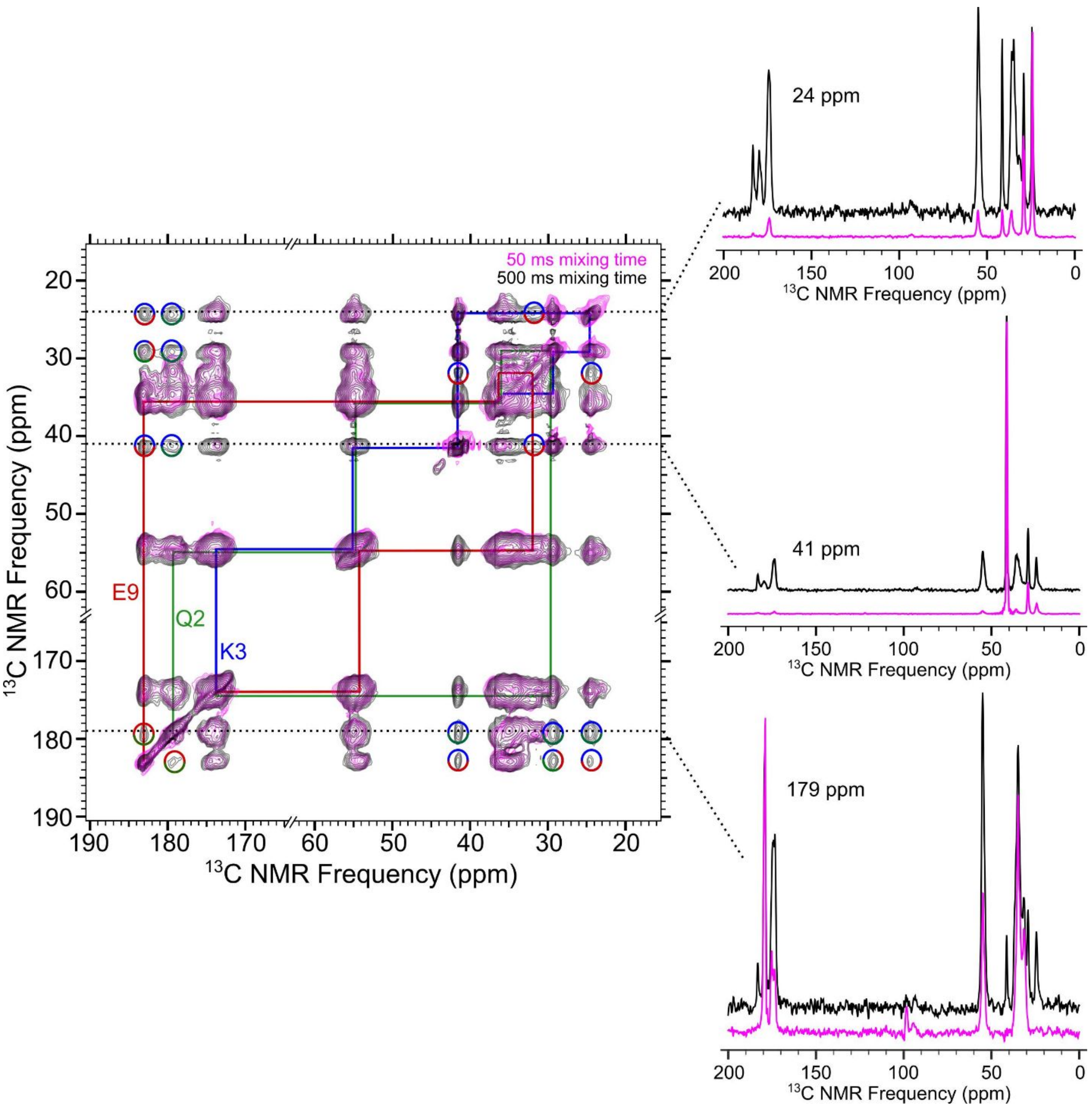

Figure S4. Overlay of 2D ^{13}C-^{13}C DARR short mixing (50 ms) and long mixing (500 ms) times of Q11 uniformly ^{13}C and ^{15}N labeled at residues Q2, K3, and E9. Multi-colored circles indicate residue contacts among the three labeled residues. 1D slices at 24 ppm, 41 ppm, and 171 ppm are shown to illustrate the ^{13}C-^{13}C couplings between residues.

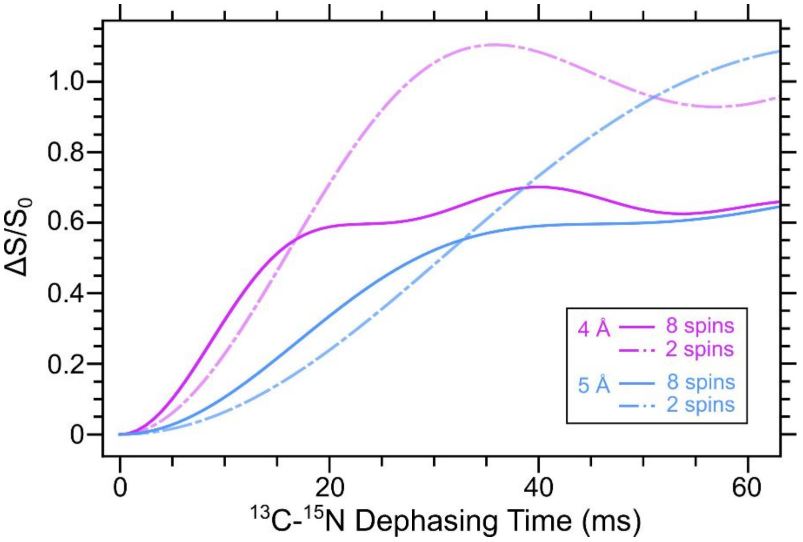

Figure S5. $^{15}N\{^{13}C\}$ REDOR spin simulation curves at 4 Å (pink) and 5 Å (light blue) comparing 2 spin simulations (dashed and dotted lines) with 8 spin simulations (solid lines).

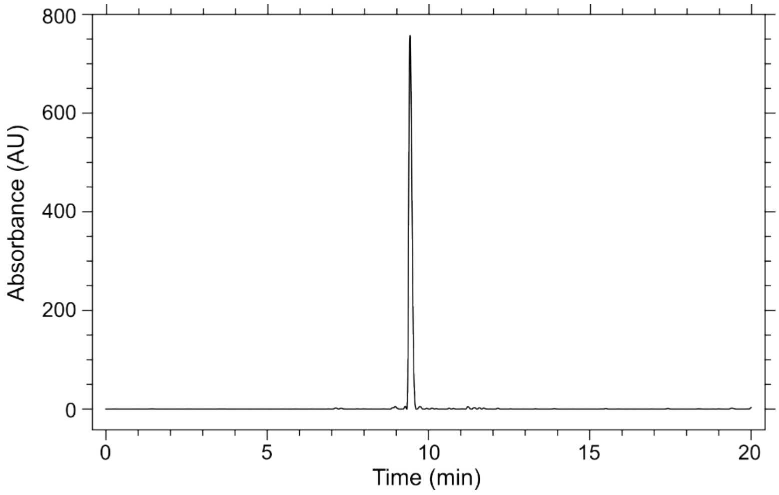

Figure S6. HPLC chromatogram of Q11 monitored at 215 nm. Estimated purity is > 95%.

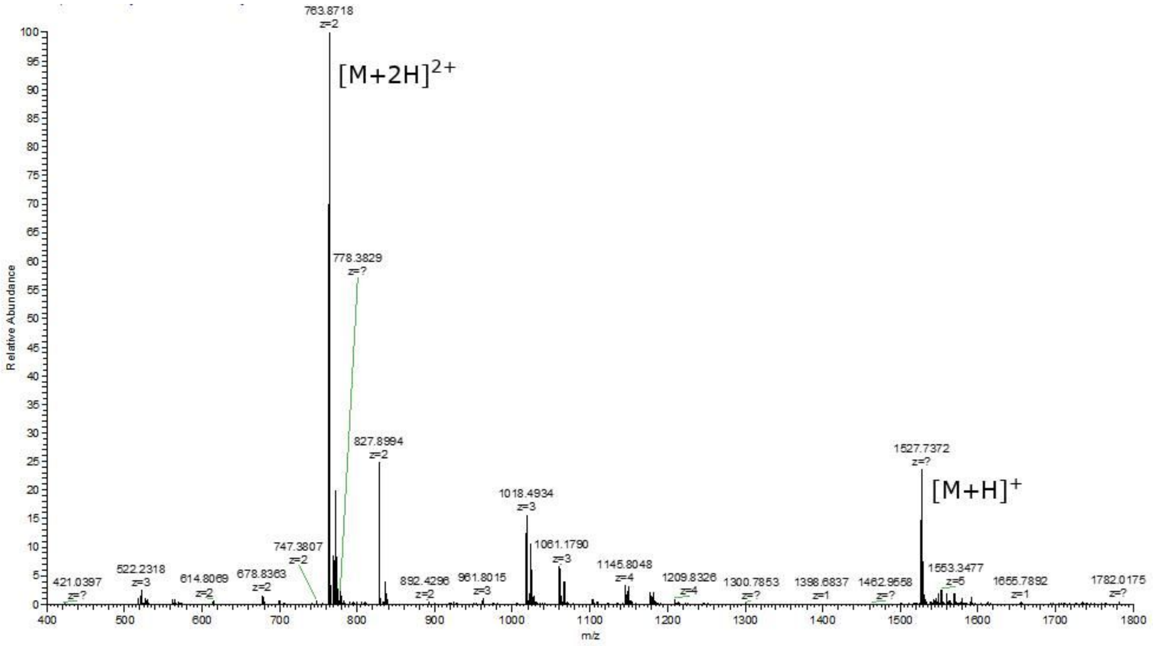

Figure S7. ESI-MS spectrum of Q11.

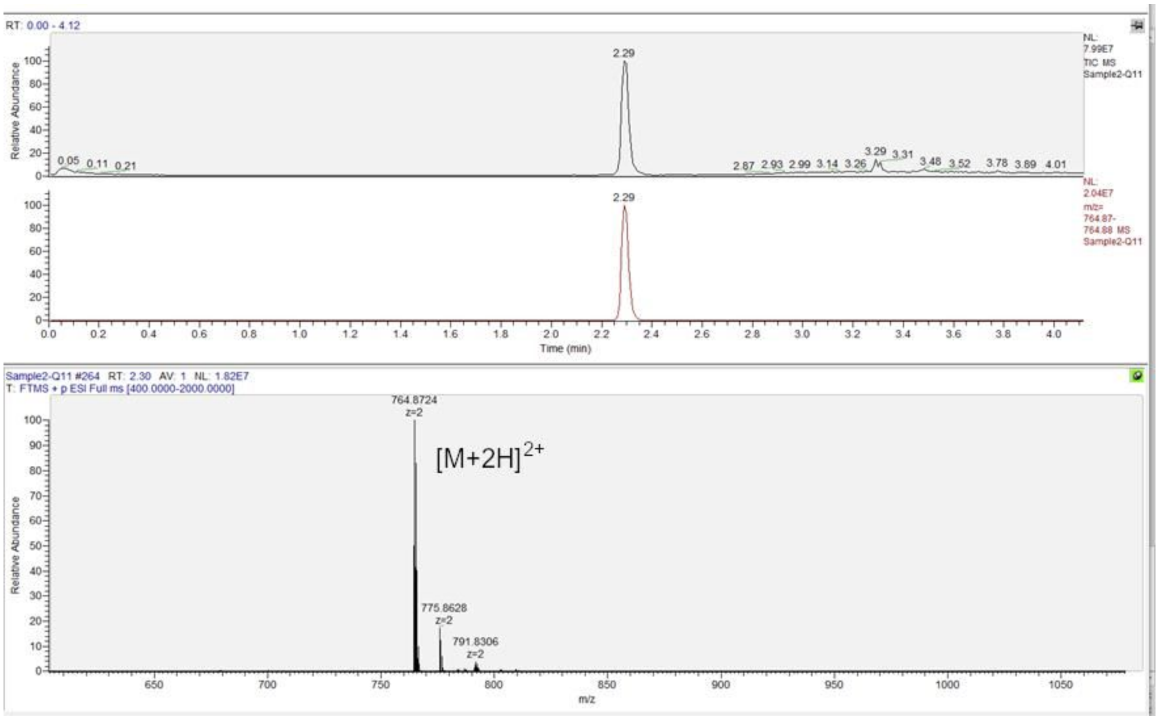

Figure S8. LC-MS spectrum of Q11 selectively labeled ^{13}CO at F4, ^{15}N at F8. Estimated purity is > 95%.

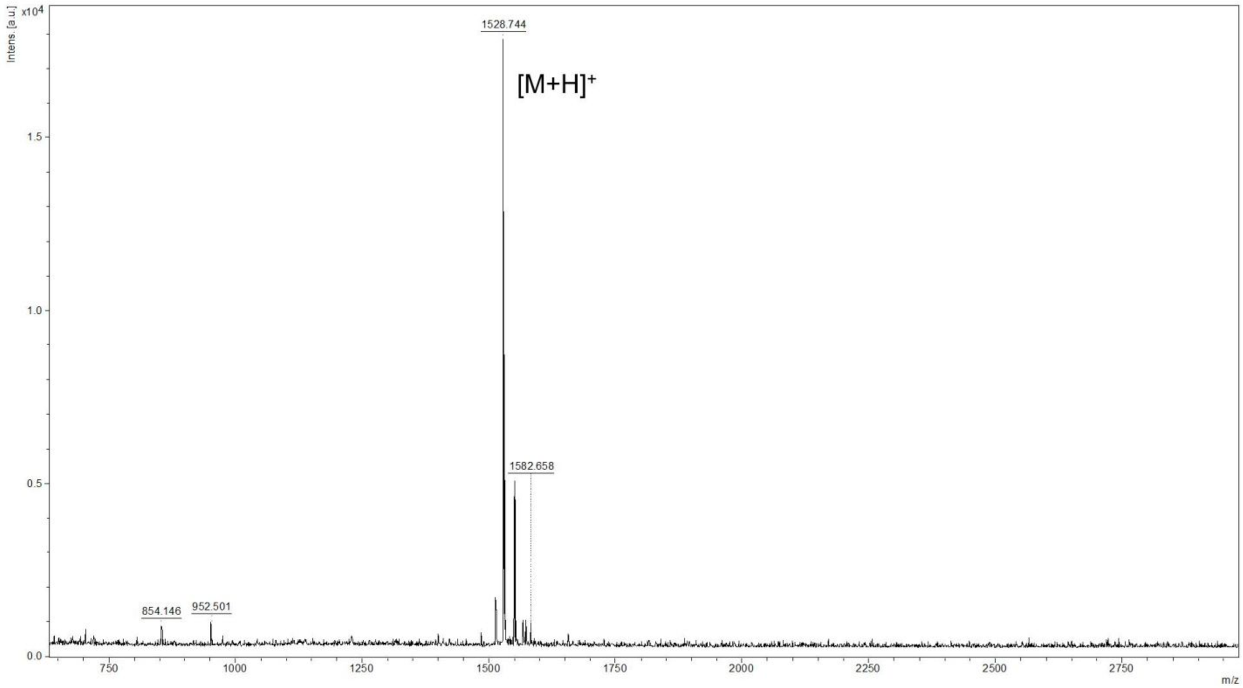

Figure S9. MALDI-MS spectrum of Q11 selectively labeled ^{13}CO at F4, ^{15}N at F8.

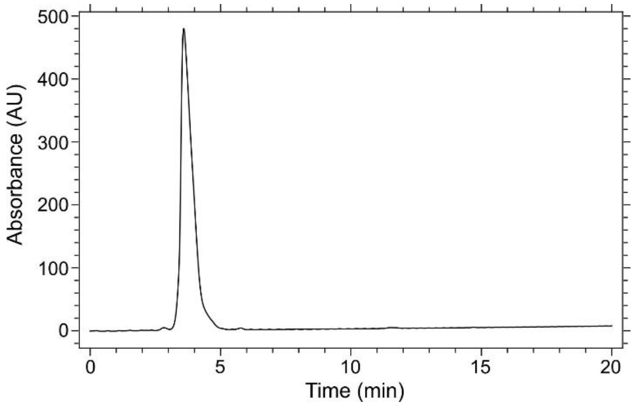

Figure S10. HPLC chromatogram of Q11 uniformly ^{13}C and ^{15}N labeled at Q2, K3, and E9 monitored at 215 nm. Estimated purity is > 95%.

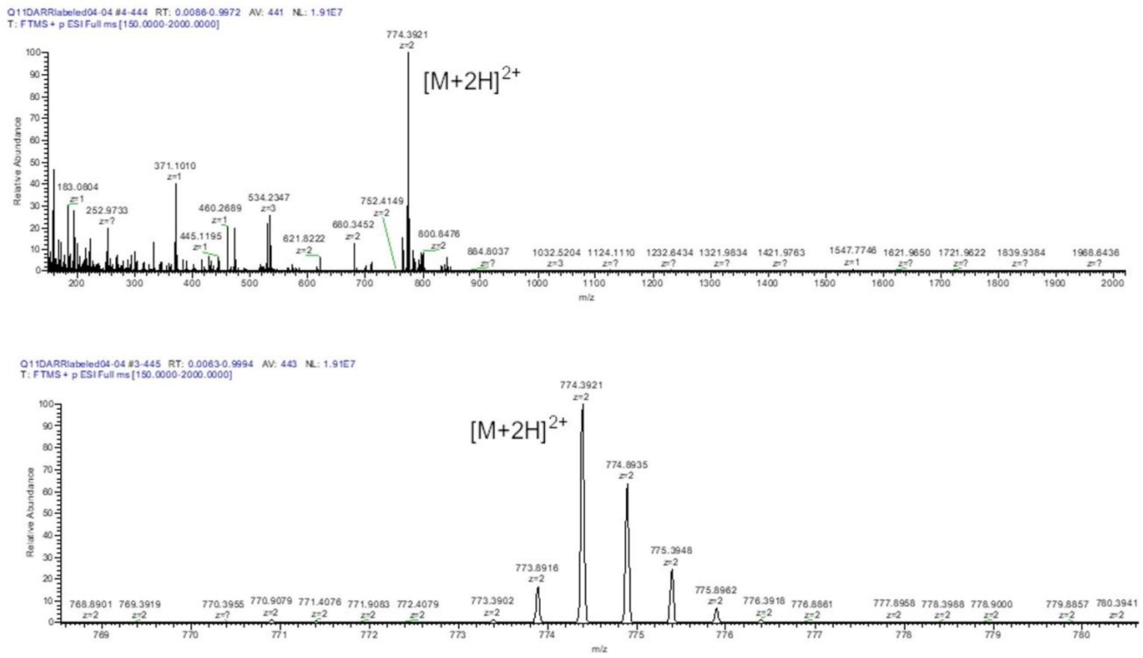

Figure S11. ESI-MS spectrum of Q11 uniformly ^{13}C and ^{15}N labeled at Q2, K3, E9.
